# Luxations

**Published:** 1845-09-01

**Authors:** 


### Luxations.

Communicated for the Buffalo Medical Journal.

At 17 years of age, being a student of medicine, I had read Cheselden's Anatomy, and was in the "13th Vol. of Van Swieten's Commentaries on Boerhave's Aphorisms," when the very respectable country physician with whom I studied was called to reduce a luxated jaw. The accident was novel to him, and making no pretensions to practical surgery, a messenger was dispatched for a surgeon some miles distant. In the interval, before the expected arrival of the surgeon, I found in our library, an ancient, odd volume on "Chirurgy", which contained directions "how to set a jaw". "On this hint I spake", and having obtained permission to attempt the reduction, I succeeded. This incident of early life determined me to become a surgeon, and though nearly half a century has gone by, in which there are few operations in any department of surgery that I have not performed with measurable success, no one has ever afforded me the pleasure, the delight, the ecstacy, of this my first essay.

"Old Luxations," are to this day, the opprobria of Surgery. Few surgeons, at this time, feel willing to undertake the reduction of a luxation that has existed 30 days.

A little girl 10 years old, suffered luxation of the hip joint forward into the foramen. The extent of the injury was not discovered until the fourth week after the accident, when a "faculty Council" of physicians and surgeons was convened, who gravely mooted the question, whether at this *late period!* it would be proper or safe to attempt reduction. A negative decision was so far over-ruled that some efforts were made to reduce it, and failing of success *at the time*, the case was abandoned. I am bound to confess that I was one of this bevy of abettors, and I cannot think of the case now without deep feelings of regret and shame.

Miss ——— a young lady of 16 years, had luxation of the hip-joint, backward on the dorsum ilii. It was thought to be a severe contusion only, until the third week; the luxation was then reduced in the usual way, without the aid of machinery.

Mr. ——— dislocated the elbow joint. The injury was mistaken for fracture of the humerus, and dressed with bandages and splints At the end of four weeks he came to me. The head of the humerus was on the forearm, and the joint anchylosed. The reduction was effected in the following manner: The body was firmly fixed; very powerful extension and rotation were made and continued, with but momentary intermission, a full hour, when reduction was accomplished. The suffering during the operation was severe indeed, and the subsequent symptoms alarming—rigors, faintings, and spasms, that yielded only after several hours of suffering and anxiety, to large and repeated doses of opium. This was an act of unnecessary cruelty which ought never to be repeated.

Luxations of the shoulder joint are the most common of luxations. Mr. R———, 22 years old, a stout and very muscular blacksmith, had luxation of the right shoulder joint into the axilla. The surgeon who dressed it assured him it was reduced. Seventy-five days after the accident he came to me; the head of the humerus was firmly fixed deep in the axilla, pressing on the axillary plexus, and causing much pain, particularly in the fingers and hand. The reduction was undertaken in the following manner: the arm was moved gently and rotated from day to day, as he could bear it, and wider and wider, until the head of the bone was disengaged from its new attachments, when extension was added to rotation for several successive days, in all I believe eight or nine days, and when the muscles seemed to be sufficiently wearied, the head of the bone was brought up to its place by a powerful effort. The glenoid *cavity* was obliterated, and the restoration of the bone "gave no sign."

The two following cases are of recent occurrence. An Irish ditcher came to me, and this is the history he gave of himself. Four months before, in excavating a sand-bank, a body of earth fell on him and dislocated his shoulder into the axilla. Dr. H., a respectable surgeon, was immediately called, and reduced the luxation without difficulty. The fourth day after the accident he went to his work, and had continued to labor since, without suffering inconvenience. On exposing his shoulder I did not hesitate, at sight, to pronounce it a luxation into the axilla. The acromion process was unusually prominent, and it needed not to be touched, I thought, to decide the nature of the injury. Of course I could not fail to feel great surprise, for I knew Dr. H. personally, and it seemed incredible that *he* should have left the shoulder in that condition; nor was my surprise abated by the fact that the patient had suffered so little from the accident, and had such full, free and unconstrained motion of the arm. Upon examination by taction, it turned out to be, not a luxation of the shoulder, but what I had not before seen, a luxation of the humeral end of the clavicle. It was, however, still a matter of amazement that Dr. H. took no measures to lessen the deformity. It happened singularly enough, that at this moment, while the patient was with me, I saw Dr. H. passing, and called him in to witness his own "handy work". The doctor looked and expressed more real, unfeigned astonishment than I felt. The condition of the joint did not admit of a doubt; but he solemnly assured me that the luxation of the clavicle did not exist when he last saw the joint. The solution is ready enough; the right shoulder was luxated, and the patient resumed his work with the spade the fourth day after the accident; the consequence was consecutive luxation of the clavicle. Except the deformity, the patient suffers nothing from the accident.

W———, a drunken Irish mendicant, 56 years old, dislocated his shoulder into the axilla. A coterie of surgeons attempted the reduction some days after the accident, and used for that purpose, what I suppose, from representation, to have been the "screw of Hippocrates." About nine weeks after the accident, reduction was undertaken by my advice in the way, substantially, described in the case of Mr. R. The head of the humerus could be felt deep in the axilla, pressing painfully on the axillary plexus. Motion, extension and rotation, were made as they could be borne, at short intervals, for a fortnight. By these means the head of the bone was brought out of the axilla, and could be felt on rotating the arm, grating on the inferior edge of the glenoid. In this position it was confidently hoped that reduction might be completely effected by a well directed and powerful effort. It was made in the following manner: the patient was laid upon the floor on his back; extension was made as before described; in addition another assistant was seated by the side of the patient, holding with both hands a firm belt passed over the luxated shoulder, and his heel in the axilla. When extension was carried to its utmost verge, the arm was rotated, and this assistant directed at the same time to draw on the belt, and force up the head of the bone with his heel. The effort did not succeed, and it was decided to wait another trial. The axillary glands, however, took on painful inflammation, which precluded all further attempts for a time, and the patient declaring himself satisfied with the relief he had obtained, could not be persuaded to submit to another trial. The deformity was comparatively slight, and no doubt was entertained that for usefulness, the arm would be ultimately well restored. The last attempt at reduction was made about the 1st of July ultimo. On the 3rd August, this miserable man, who had been in a state of daily inebriation for several weeks, fell in the street and instantly expired. Five hours after death the shoulder was examined. An incision was made from the neck over the acromion process, and down the arm below the insertion of the deltoid muscle. In laying off the integuments the attachments of the deltoid and pectoral muscles were severed, and the capsular ligament fairly exposed. In this stage of the examination attempts were made to raise the bone to its place, but they were unsuccessful. The capsule was next divided laterally, laying open the glenoid and showing the head of the humerus resting against the inferior edge. The glenoid was filled with cartilaginous deposit in which were detected some spiculæ of bone. An attempt was now again made to bring up the head of the bone, but it resisted our efforts. Lastly the attachment of what I took to be the sub-scapularis muscle was divided, and the bone was readily brought to its place.

In my life, which has not been a brief one, and with a practice not very limited, I have never failed to reduce a recent luxation of the shoulder joint. My position and relations have been such that I have often been refered to by my less fortunate professional friends, and with equal success; nor do I recollect any serious ill-consequences to have followed the operation. I owe this, no doubt, to the manner of reduction, and I hope you will pardon my egotism and garrulity, in formally describing it. To reduce a luxation of the shoulder joint, the surgeon will need four trusty assistants. If the luxation be into the axilla, seat the patient on the floor, and fix the body by passing a broad belt or band around it under the dislocated arm, and brought over and held firmly by an assistant, on the processus acromion of the sound side. Wrap a damp napkin around the arm above the elbow and loop over it a suitable strap (or silk handkerchief) to be used for extension; an assistant grasps the arm above the elbow, passing his hand underneath it with one hand, while he takes the arm at the wrist in the other. Bending the arm to a right angle, he raises the elbow *above* a right angle and parallel with the body, *and as much above* as is consistent with the necessary extension, thus removing all resistance from the deltoid and supra spinatus muscles. Another assistant now inserts his hands into the loop of the strap or handkerchief, one on the outside, the other passed under the elbow on the inside. The fourth assistant stands behind "a corps de reserve." The surgeon, standing behind the patient, grasps the shoulder joint with both hands, thrusting the fingers of each deep into the axilla behind the head of the bone, with his thumbs resting (if he can reach it) on the acromion process, directs extension to be made deliberately and forcibly, follows with his fingers the head of the bone as it emerges from the axilla, and when it approaches the inferior edge of the glenoid directs the supernumerary assistant to grasp the arm with both hands above the loop, and while *he* adds to the force of extension, the *first* assistant is directed to rotate the limb; the surgeon at the same time raises the head of the bone with his fingers, and it slides into its place with a loud report. If the luxation be forward, under the pectoral muscle, the arm should be inclined backward in making extension; if it be backward on the scapula, which I have seen but twice (in the same individual) the arm should be inclined forward. I use no machinery. Years ago I was prompted to use the "pullies", but I found them sadly in my way and put them aside. F.

*July* 1845.

